# Tissue adaptation of CD4 T lymphocytes in homeostasis and cancer

**DOI:** 10.3389/fimmu.2024.1379376

**Published:** 2024-04-16

**Authors:** Marina V. A. Pereira, Rômulo G. Galvani, Triciana Gonçalves-Silva, Zilton Farias Meira de Vasconcelo, Adriana Bonomo

**Affiliations:** ^1^ Laboratory on Thymus Research, Oswaldo Cruz Institute, Oswaldo Cruz Foundation, Rio de Janeiro, Brazil; ^2^ Laboratory of High Complexity, Fernandes Figueira National Institute for The Health of Mother, Child, and Adolescent, Oswaldo Cruz Foundation, Rio de Janeiro, Brazil; ^3^ National Center for Structural Biology and Bioimaging - CENABIO, Federal University of Rio de Janeiro, Rio de Janeiro, Brazil

**Keywords:** CD4 T lymphocytes, tissue-specific immune response, tumor immunology, T CD4 tumor response, cancer

## Abstract

The immune system is traditionally classified as a defense system that can discriminate between self and non-self or dangerous and non-dangerous situations, unleashing a tolerogenic reaction or immune response. These activities are mainly coordinated by the interaction between innate and adaptive cells that act together to eliminate harmful stimuli and keep tissue healthy. However, healthy tissue is not always the end point of an immune response. Much evidence has been accumulated over the years, showing that the immune system has complex, diversified, and integrated functions that converge to maintaining tissue homeostasis, even in the absence of aggression, interacting with the tissue cells and allowing the functional maintenance of that tissue. One of the main cells known for their function in helping the immune response through the production of cytokines is CD4^+^ T lymphocytes. The cytokines produced by the different subtypes act not only on immune cells but also on tissue cells. Considering that tissues have specific mediators in their architecture, it is plausible that the presence and frequency of CD4^+^ T lymphocytes of specific subtypes (Th1, Th2, Th17, and others) maintain tissue homeostasis. In situations where homeostasis is disrupted, such as infections, allergies, inflammatory processes, and cancer, local CD4^+^ T lymphocytes respond to this disruption and, as in the healthy tissue, towards the equilibrium of tissue dynamics. CD4^+^ T lymphocytes can be manipulated by tumor cells to promote tumor development and metastasis, making them a prognostic factor in various types of cancer. Therefore, understanding the function of tissue-specific CD4^+^ T lymphocytes is essential in developing new strategies for treating tissue-specific diseases, as occurs in cancer. In this context, this article reviews the evidence for this hypothesis regarding the phenotypes and functions of CD4^+^ T lymphocytes and compares their contribution to maintaining tissue homeostasis in different organs in a steady state and during tumor progression.

## Introduction

Immune cells are present in different organs, which are believed to promote local immune surveillance. The tissue compartmentalization of the Immune System (IS) is essential for the response against pathogens and local injuries and critical in tissue homeostasis-controlling processes such as angiogenesis, morphogenesis, and tissue repair (1–3). However, given the particularities of each tissue, it makes sense that the activities of IS cells will vary depending on the host organ. Different tissues provide a singular microenvironment for the resident or migrating immune cells. Also, different challenges are met in each organ and represented by the pathogen/pathology that targets each tissue.

Although blood is a valuable biological sample rich in IS cells, most of these cells reside and perform their functions within various tissues. Specifically, in T cells, blood samples comprise only a small portion, approximately 2 to 3%, of the total lymphocyte population. Most T cells are distributed throughout lymphoid organs such as the bone marrow, spleen, and lymph nodes and within barrier surfaces like the skin, gut, and mucous membranes (4). Moreover, it has already been demonstrated that CD4^+^ T lymphocytes, in particular, may reside in environments previously considered immunologically privileged (5). The wide tissue distribution of T lymphocytes reflects their importance, going beyond simple immunosurveillance, but surveillance influenced by the host tissue and with a possible role in tissue homeostasis.

It is well known that CD4^+^ T lymphocytes are central controllers of the immune response. Through cell-to-cell contact and the secretion of specific molecules, they modulate several components of the IS and are important determinants of the final fate of an immune response (6). The ontogeny of T lymphocytes is marked by a rigorous maturation process, where their future functionality is tested. During its development, progenitors committed to the lymphoid lineage are generated in the bone marrow and migrate to the thymus, where they will be selected according to their “fitness” to the host. At the end of the process, the chosen repertoire will ideally be perfect for the specific individual host, with the best fitness and minimal potential damage to their host (7–10). Through maturation, surviving naive T lymphocytes migrate to the periphery and lodge in secondary lymphoid and non-lymphoid organs.

The migration of CD4 T helper (Th) lymphocytes to a specific site does not occur randomly; the process is controlled by the expression of homing molecules, such as chemokine, integrin, and selectin receptors present on the surface of lymphocytes and their ligands in the vasculature of the target site. Thus, through the expression of CD62L, L-selectin, and CCR7, T cells enter the lymph nodes (LN). In contrast, the expression of α4β7 and CCR9 guide their homing to the small intestine, while CCR4 is associated with the migration of T cells to the skin during inflammatory processes (11, 12). So, migration and parking of T cells in different sites are guided by a specific group of chemokines and their receptors, which define the T cells’ address. This definition is instructed at the T cell activation site, the draining lymph node of the affected site, where antigen-presenting cells and stromal cells address the T cells that are recognizing MHC+peptide complex (MHCp) to the same site where the antigen was picked up and fixed by the tissue to which the lymphocyte was sent (13). However, this is not that strict for specialized tissues and can be shared, for example, by mucosal sites such as the intestines and respiratory mucosa (14). In addition to factors such as antigen concentration, antigen-presenting cell (APC) type, and costimulation, the final Th cell fate is defined by the specific *milieu* of cytokines present during the activation process, leading to well-established effector profiles. Th cells, such as Th1, Th2, Th17, or Regulatory T cell (Treg), amongst others, can produce a range of different cytokines, which classifies their phenotype.

The differentiation of CD4^+^ T lymphocytes into the Th1 profile is governed by signaling through IL-12 and gamma interferon (IFN-γ), activating the T-box transcription factor (T-bet) (15) and leading to the production of tumor necrosis factor-alpha (TNF-α), IL-2, and IFN-γ (16, 17), crucial for combating intracellular pathogens (18, 19). Conversely, the Th2 phenotype, regulated by GATA-binding protein 3 (GATA3) and induced by IL-4, orchestrates responses against extracellular pathogens, orchestrating effector cytokines like IL-4, IL-5, IL-9, and IL-13 (20, 21), while recruiting eosinophils, basophils, and mast cells, significant cells in helminth infections and allergic reactions (22–24). The versatility of CD4^+^ T lymphocytes is exemplified by the Th17 phenotype, mediated by retinoic acid receptor-related orphan gamma-T receptor (RORγt), requiring cytokines such as transforming growth factor beta (TGF-β) and IL-6 (25, 26), crucial in autoimmune diseases and immune responses against extracellular bacteria and fungi (27–30).

Additionally, CD4^+^ T lymphocytes exhibit a regulatory response represented by Treg phenotype, involving transcription factor forkhead box P3 (FOXP3) and TGF-β, crucial for immune homeostasis and autoimmunity prevention through secretion of anti-inflammatory cytokines like IL-10 and TGF-β (31–33). Other CD4^+^ T lymphocytes subsets like Follicular T helper (Tfh), Th9, and Th22 cells exhibit distinct functions, contributing to antibody-mediated responses, allergic diseases, and tissue repair (34–36). The plasticity of CD4^+^ T lymphocytes allows for diverse functional phenotypes beyond traditional subsets, with Th17 cells capable of transitioning into Th1-like cells or Tregs, underscoring their dynamic nature and ability to respond to tissue-specific cytokine milieus (37–41).

The plasticity of T lymphocytes has important implications in disease contexts, especially in cancer. The frequency or presence of these lymphocytes can have conflicting results on the prognosis of tumor progression and response to treatments, including immunotherapy (**Table 1**). This is different for various types of tumors, indicating that different phenotypes of CD4^+^ T lymphocytes play roles in shaping the tumor microenvironment (TME) (83, 84). Therefore, it is important to further explore the role of CD4 T lymphocytes in immune regulation and effective responses in specific environments. This emphasizes their potential as targets for organ-specific anti-tumor therapies (85).

**Table 1:** Prognostic value of lymphocytes infiltrates across tumor types in tumor progression and immunotherapy.

Organ	Cancer type	Tumor Infiltrate Lymphocytes	Prognostic value	Reference
Skin	Melanoma	Total lymphocytes	Positive	(42–44)
			No Association	(45–47)
			Negative	(48)
		CD4	Positive	(43, 44, 49–51)
			No Association	(47)
		CD8	Positive	(43, 44, 49, 50, 52–54)
			No Association	(47, 55)
Lung	NSCLC	CD4	Positive	(56–59)
			No association	(60, 61)
		CD8	Positive	(58, 59, 62–64)
			No Association	(57, 60)
			Negative	(56)
Liver	HCC	CD4	Positive	(65–67)
			Negative	(68, 69)
		CD8	Positive	(65, 70–72)
			No Association	(73)
			Negative	(67)
Gut	CRC	CD4	Positive	(74, 75)
			No Association	(76)
			Negative	(77)
		CD8	Positive	(78–80)
			No Association	(81, 82)

### The balance of lung T cell phenotypes profoundly impacts tissue maintenance and tumorigenesis

#### Pulmonary Tregs act as guardians, preventing overactivation of the Th2 response, while resident memory T cells exhibit a diverse profile

The human lung has an estimated area of approximately 70 m^2^ and is continuously exposed to the external environment (86). They have a complex architecture formed by branching airways and alveoli parenchyma that comprise an extensive surface area to maximize the gas exchange. Therefore, the lung microenvironment deals with different antigenic stimuli requiring an intricate and complex cellular community to maintain homeostasis. Among the cellular components, immune cells, including dendritic cells, resident alveolar and interstitial macrophages, and T and B lymphocytes, are crucial players in homeostatic and pathological conditions (86–90).

Although most lung development occurs during the embryonic period, this organ is not fully formed at birth. The formation and maturation of the alveoli in rats and mice occur postnatally, whereas, in humans, it begins just before birth but continues postnatally (91). Domingo-Gonzalez and colleagues showed that mice’s lung changes after birth were induced by the transition from a fluid hypoxic environment in the embryonic phase to an air/oxygen environment, affecting the immune cell population in this site (92). In addition, most lung T lymphocytes in neonatal mice display a CD4^-^CD8^-^ phenotype early in life. Still, most of these cells exhibited Trac expression, identifying them as conventional T cells (αβ T cells) rather than γδ T cells. CD4^+^ T cells increase over time, are more numerous than CD8^+^ T lymphocytes, and produce IL-4 (Th2 phenotype). In parallel, lung colonization by microbiota also occurs and modulates CD4^+^ lymphocyte populations at this site after birth. Curiously, in mice, in just two weeks postnatally, the species of the bacterial population changes in the lungs and induces upregulation in PD-L1 expression on lung CD11b^+^ dendritic cells, which are responsible for differentiation of peripheral Treg. These cells are crucial to controlling an exaggerated Th2 microenvironmental and aeroallergen responsiveness and are maintained in the adult lung, which is essential in keeping a healthy lung. Corroborating these facts, germ-free mice mount exaggerated Th2 allergic airway inflammation, and this crosstalk between microbiota lung colonization after birth with the immune system maturation/modulation in this microenvironment has also been observed in humans (93–95).

In healthy mature pulmonary human tissue, CD4^+^ T lymphocytes are found in greater frequency when compared to CD8^+^ T lymphocytes. This population is composed of naive and, mostly, memory CD4^+^ T lymphocyte phenotypes. The latter, characterized by the expression of CD69^+^ and CD103^+^ (CD103 expression to a lesser extent and more heterogeneous), showed a particular tropism for the pulmonary tissue and remains retained in the lungs even in the absence of antigen or inflammation (96–98). Indeed, analysis of bronchoalveolar lavage obtained from lung transplanted patients in the first year after transplant showed that CD4^+^ T lymphocytes from the donor persisted in this allograft, reinforcing their residency within the tissue. These cells exhibited the canonical phenotype of memory CD4^+^ CD103^+^ CD69^+^ and produced IFN-ɣ, IL-2, and IL-17 upon in-vitro activation (99). Accurate analysis of single-cell confirmed that in healthy human lungs, CD4^+^ T lymphocytes displayed mostly a tissue-resident memory phenotype enriched for the expressions of genes related to Th17 (CL20, RORA, RORC, IL17A) and Treg (FOXP3, TIGIT, CTLA4, IL2RA) subtypes, and express characteristic integrins and chemokine receptors (ITGA1, ITGAE, VIM, CXCR6) (100). Along with their traditional role in maintaining immunological tolerance and limiting immune responses, lung CD4^+^ Tregs are also associated with pulmonary tissue repair after a homeostatic breakdown.

**Figure 1:**
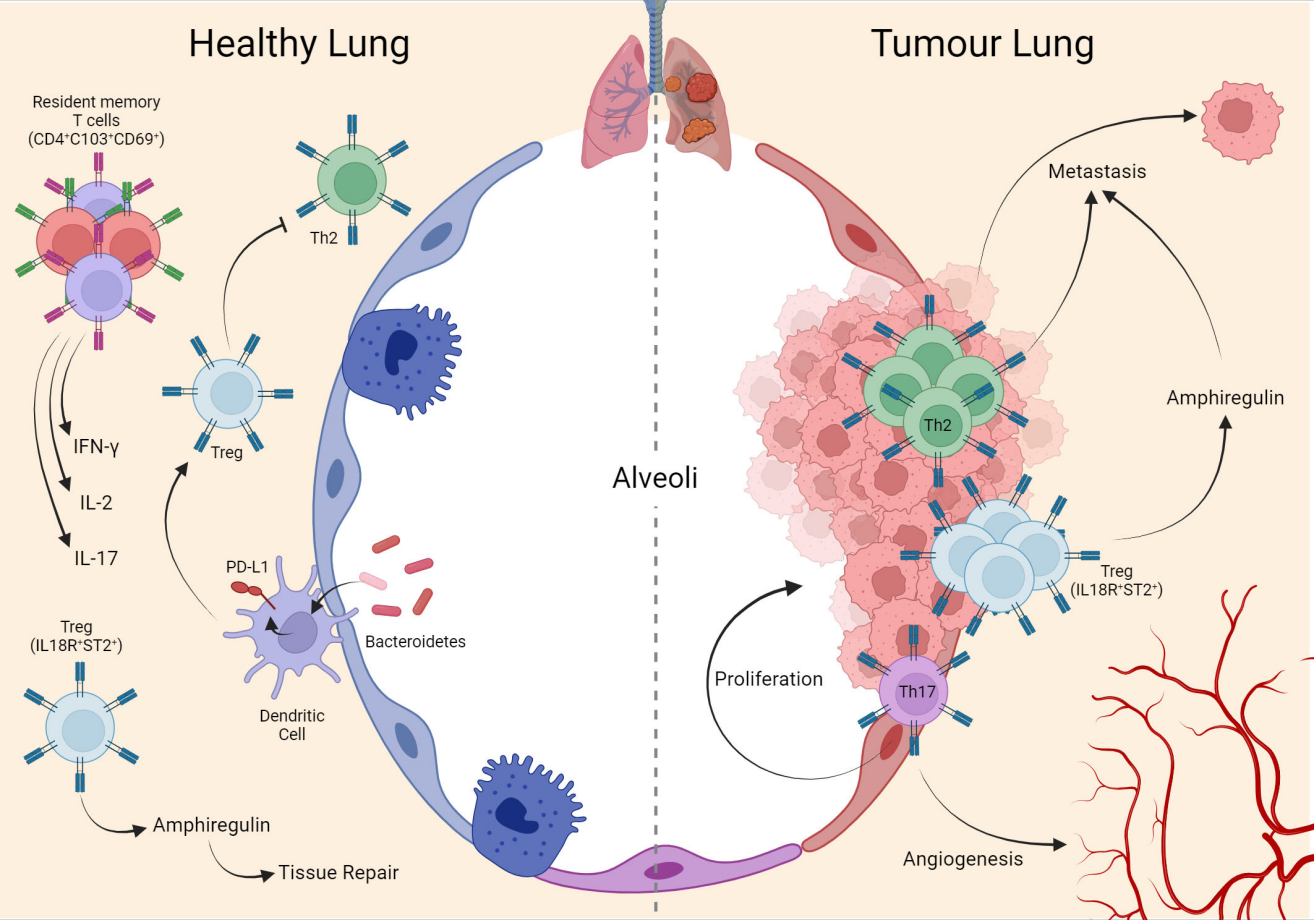
Interaction of T cells in lung homeostasis and cancer. During lung homeostasis in mice, the local microbiota induces PD-L1 in dendritic cells which support the generation of local Treg cells, important for suppressing the Th2 phenotype that is exacerbated in the tissue in the postnatal period. Another population of Treg (IL18R^+^/ST2^+^) assists tissue repair through the production of amphiregulin. In adulthood, mature human lung tissue is composed of memory T cells (CD4^+^ CD103^+^ CD69^+^) expressing IFN-ɣ, IL-2, and IL-17. These same phenotypes remain during the establishment of the tumor in human lung tissue, with emphasis on Th17 supporting tumor cell proliferation and angiogenesis. Mouse metastasis data demonstrate that Th2 and Treg (IL18R^+^/ST2^+^) are important for lung-specific metastasis in breast tumors. Created with BioRender.com.

Besides the presence of effector CD4^+^ T lymphocytes, the lungs harbor a distinct population of Treg cells expressing high levels of IL18R and ST2^+^ (IL-33 receptor), which, independent of TCR engagement, produce amphiregulin, a molecule involved in tissue repair. In addition, in the absence of Treg-derived amphiregulin, a significant decline in lung functions was observed in a murine model of influenza virus infection, with no impact on antiviral immune response nor in viral load (101). This observation indicates a vital role of Treg in keeping lung integrity independent of immune regulation, and separate cues invoke these functions. A similar role for Tregs had been described in muscle regeneration, although Treg specificity was central for homeostatic tissue control (**Figure 1**) (102, 103).

#### Th17 induces lung tumor proliferation and angiogenesis, while Th2 and Treg promote metastasis

As an essential component of the pulmonary microenvironment and, due to their plasticity and intrinsic characteristics of coordinating the immune response, CD4^+^ T lymphocytes are responsive to changes in this site, which are important during cancer. Primary lung tumor is a heterogeneous disease with a wide range of clinicopathological features. They are classified broadly as non-small-cell lung cancer (NSCLC - 85% of total diagnoses) or small-cell lung cancer (SCLC - 15% of total diagnoses) (104). There is little evidence about the influence of infiltrated CD4^+^ T lymphocytes at the SCLC site. Still, poor survival has been correlated with a high proportion of intratumoral Treg lymphocytes (ratio FOXP3^+^:CD45^+^) (105).

A screening of immune cell composition in NSCLC demonstrated that CD3^+^ T cells constituted nearly half of all tumor-infiltrating CD45^+^ leukocytes. Among these T lymphocytes, CD4^+^ T lymphocytes represent the most abundant population and exhibit a memory/effector phenotype (106). Looking into CD4^+^ T lymphocytes subpopulations, it was observed an evident Th2 and Treg cell predominance in NSCLC stroma and tumor epithelium, with lower numbers of Th1, Th17, and Tfh (107, 108). A systematic meta-analysis revealed that total CD4^+^ T lymphocytes in the tumor stroma compartment were associated with improved overall survival. In contrast, the presence of Treg cells in the tumor stroma was linked to a poorer prognosis (109). In line with these results, high Treg cell islets in NSCLC were associated with worse survival (110). In another cohort analysis, although CD4^+^ infiltration into NSCLC was associated with advanced tumor stages and lymph node spread, high CD8^+^ frequency in TILs was related to improvement in overall survival (111).

As seen in NSCLC human patients, in the mouse model of lung tumor (CCSP^cre^/K-ras^G12D^), a robust infiltration of Treg as well as Th17 cells, but not of Th1 lymphocytes in the tumor tissue was shown (in this paper, the presence of Th2 was not investigated). Indeed, these cells were essential players during tumor development, given that IL17-/- animals exhibited a 75% reduction in tumor numbers compared to wild-type mice. This effect has been attributed to Th17 cells, which secrete IL17, which in turn acts on the TME by inducing the expression of G-CSF and the recruitment of immunomodulatory myeloid cells that produce not only VEGF, but also Bv8, which also has an angiogenic role. In fact, this explains the resistance to anti-VEGF therapy in individuals with Th17 cell infiltration (112–114). Adding to the role of IL-17 as a tumor-promoting cytokine, Salazar Y. and colleagues demonstrated in humans that a high frequency of Th9 or Th17 among CD4^+^ T lymphocytes was significantly associated with decreased survival in NSCLC patients. This was associated with increased EMT induced by both cytokines in both *in vivo* and *in vitro* models (115).

Tumor burden causes changes in lung homeostasis, which was also observed in the profile of NSCLC-infiltrating Treg cells. These cells have specific expression patterns compared to circulating Treg cells and those from normal lungs. Tumor-infiltrating Treg cells were highly suppressive, upregulate immune-checkpoint-related molecules, and showed particular signatures regarding surface molecules such as IL1R2, PD-L1, PD-L2, and CCR8, suggesting that the NSCLC microenvironment influences Treg cells profile and functionality (116). Intratumoral Treg cells produce high amounts of amphiregulin, which promotes tumor growth without interfering with tumor immune status, probably through the exact mechanism described for lung tissue repair (101, 117).

Besides their influence in primary lung tumors, CD4^+^ T lymphocytes are also implicated in modulating the lung microenvironment for metastatic colonization. In the *PymT* murine breast cancer model, it was demonstrated that Th2 cells are crucial to induce lung metastasis. These cells produce IL-4 that modulates macrophages into a protumorigenic phenotype, producing an epithelial growth factor that stimulates local tumor growth. Metastasis in this model was abolished in the RAG1-/- background and significantly reduced in PyMT/IL4Ra^-/-^ mice (83). Related to Treg phenotype, in a murine breast cancer model (4T1), intranasal treatment with rmIL-33 induces an increase in Treg IL-18R^+^ ST2^+^ amphiregulin in the lungs. These Tregs were directly associated with elevated metastatic lung burden in treated mice compared to controls and were entirely amphiregulin-dependent (118).

The evidence gathered here supports that CD4^+^ T lymphocytes are natural and essential components of lung tissue and can exert their influence directly and indirectly in different contexts. This is related to their remarkable plasticity and phenotypic diversity in a microenvironment exposed daily to many antigenic challenges. Through reciprocal interactions, these cells play essential roles in maintaining a tolerogenic environment, limiting infections, and maintaining homeostasis and tissue repair. In pathological situations like tumor development, which happens slowly inside the organ, homeostatic adaptations may cause changes in CD4^+^ T lymphocyte composition. These alterations exacerbate or unbalance homeostatic functions, albeit assuming pro or anti-tumorigenic roles. Recognition of the different T cell phenotypes will be critical in a therapeutic approach (**Figure 1**).

### T cell role within the bone: regulation of hematopoiesis, bone remodeling and involvement in bone tumors

The bone is a complex structure that houses two systems: the bone itself, as part of the skeletal system, and the bone marrow (BM), as part of the hematopoietic system. These systems cohabit closely and influence each other (119, 120). T cells represent about 2-5% of total BM nucleated cells in this microenvironment. Moreover, BM exhibits a reduced CD4^+^/CD8^+^ ratio compared to the 2:1 observed in peripheral blood and shows a high proportion of memory T cells even without a defined cognate antigen (121). These memory cells are believed to depend primarily on IL-7 and IL-15, which are rich in the environment. Furthermore, one-third of all CD4^+^ T lymphocytes in this microenvironment are Treg cells (122–124). Despite representing a small population in this microenvironment, CD4^+^ T lymphocytes are essential players in both compartments.

#### T cells help the bone remodeling system in health

The bone is a dynamic tissue that is in a constant remodeling process. It is estimated that in adults, approximately 10% of all bone mass is renewed yearly. Bone metabolism is controlled by the interaction of many different cells and diverse signaling pathways that converge directly or indirectly. This intricate process is organized by a balance between osteoclasts, which are responsible for bone resorption, and osteoblasts, which, on the contrary, promote bone formation. At the molecular level, the signaling axis involving the Receptor activator of NF-kB (RANK), the Receptor activator of NF-kB ligand (RANKL), and Osteoprotegerin (OPG) is crucial. By binding to RANK, expressed in osteoclast precursors, RANKL induces mature osteoclasts’ formation, activation, and survival. This process is counterbalanced by OPG, which acts as a decoy receptor for RANKL and prevents it from binding to RANK, inhibiting osteoclastogenesis (125).

The involvement of T cells in bone metabolism began with the observation that T-cell-deficient mice exhibit alterations in bone parameters (126, 127). Juvenile athymic mice (4 weeks old) exhibit average bone mineral density; in contrast, adult athymic mice (12 – 16 weeks old) show a reduction in bone mineral density and bone volume compared to the wild-type partner (127, 128). It has been demonstrated that B lymphocytes are a necessary source of OPG, which is T cell-dependent through CD40L-CD40 interaction. In agreement, CD40L knockout mice display significantly lower concentrations of OPG in bone marrow, decreased bone mineral density, and total bone mass relative to their respective wild type (127). The adoptive transfer experiments also showed evidence of the CD4^+^ T lymphocytes impact on bone turnover. Purified CD4^+^ T lymphocytes transferred into TCRβ KO mice induce a significant decline in bone mineral density and total bone mass, which wasn’t observed if CD8^+^ T lymphocytes were the cells transferred. So, the osteoclastogenic potential was significantly higher in CD4^+^ than in CD8^+^ T lymphocytes (129).

**Figure 2:**
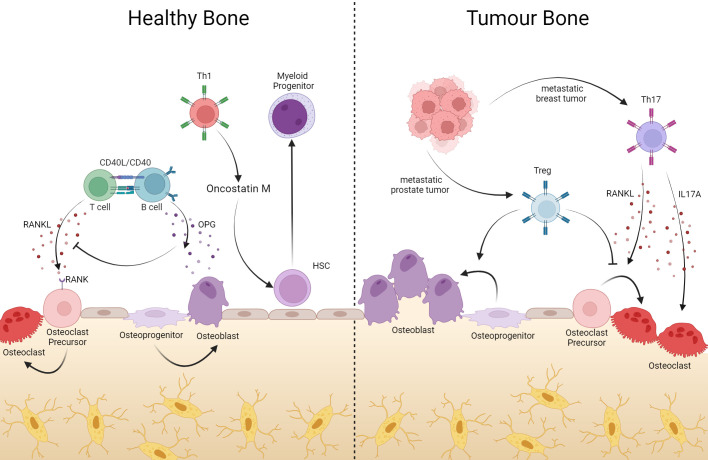
In bone, T cells help with bone remodeling, but they can also support tumor processes. Bone resident T cells produce RANKL which, once bound to its RANK receptor on the surface of osteoclate precursors, induces the maturation of these cells, contributing to the composition of the bone matrix. Via CD40L-CD40 interaction, B cells produce OPG, inhibiting RANK-RANKL binding with consequent blockage of osteoclastogenesis. In addition to bone remodeling, CD4^+^ T lymphocytes act in hematopoiesis, where Oncotain M-producing Th1 cells induce HSC differentiation into myeloid progenitor. However, in the event of tumors or bone metastases, Th17 cells producing RANKL induce osteoclastogenesis, generating bone consumption in favor of the tumor. On the other hand, Treg cells can suppress differentiation into osteoclasts and assist in the differentiation of osteoblasts. Created with BioRender.com.

Among CD4^+^ T lymphocytes subsets, Th17 and Treg lymphocytes stand out for their important contribution to maintaining bone homeostasis, especially in osteoclast differentiation. It has already been shown that CD4^+^ CD25^+^ FOXP3^+^ T regulatory lymphocytes impair osteoclast differentiation *in vitro*, mainly by cell-cell contact via CTLA-4 and secretion of anti-osteoclastogenic cytokine (such as IL-4, IL-10, and TGF-β) (130–132). In line, scurfy mice (deficient in regulatory T cells) exhibit a significant volume reduction in both trabecular and cortical bone and have an elevated number of osteoclasts per bone surface area (133). Another important link between CD4^+^ T lymphocytes and bone metabolism is that these cells express and secrete RANKL, which acts directly on osteoclastogenesis and osteoclast functionality (84). The group led by Choi Y. was a pioneer in demonstrating that T lymphocytes express RANKL. However, by that time, the relevance of T cell-derived RANKL was studied in T–dendritic cell interaction (134–136). In that same period, the molecule that regulates osteoclast differentiation was identified as RANKL (137, 138). In addition, Th17 expresses high levels of RANKL when compared to Th1 and Th2. However, their model did not show the direct role of this T cell-derived RANKL in bone consumption, where they claim IL-17 would be the critical T cell-derived osteoclastogenic mediator (139, 140). Another vital evidence regarding the role of T cells in bone homeostasis is the osteopetrosis phenotype of germ-free animals, which is corrected after T cell activation (**Figure 2**) (141, 142).

#### T cells are required in the bone marrow in homeostatic conditions or under demand

In this same microenvironment, the BM maintains and differentiates adult hematopoietic cells. These hematopoietic cells compose all mature peripheral blood cells with variable life spans. Hematopoietic progenitors undergo a constant and continuous replenishment throughout the lifetime of an organism to supply the peripheral demand. Derived from multipotent stem cells, hematopoietic cells represent a heterogeneous group with particular morphological characteristics and various functions, which include erythrocytes, megakaryocytes, myeloid and lymphoid lineages. The myeloid and lymphoid lineages are classified as part of the immune system (143, 144).

The BM architecture is categorized in a specialized microenvironment composed of different cell types that together create, through cell-cell contact, extracellular matrix, and soluble factors, a niche that can regulate the hematopoietic stem cell, maintaining its self-renewal and the balance between quiescence and lineage commitment, that englobes cell fate determination, proliferation and maturation into the hematopoietic lineages, called hematopoietic stem cell niche (145–147).

Observations from hematopoietic stem cell transplantation studies suggest an important role of T lymphocytes in hematopoiesis. Although T cells are associated with the risk of developing graft versus host disease, these cells are also related to the success of hematopoietic cells grafting in BM besides the graft versus malignancy effect. Indeed, the number of CD3^+^ T cells in the donor-mobilized hematopoietic cells inoculum is inversely related to graft failure (148). In agreement, hematopoietic grafts depleted of T cells induce less graft versus host disease, but these patients exhibit critical engraftment impairment and higher relapse rates (149–151).

Detailed analysis of BM samples from patients after allogeneic hematopoietic stem cell transplantation showed a notable difference between patients with poor engraftment and those with good graft function. In the first group, there was a significant increase in Th1 and Th17 subsets of T lymphocytes. Also, Th2 cells were fewer in patients with poor graft function. No significant difference was observed when comparing Treg cells between these groups. However, the ratio of Th17/Treg was higher in patients with poor graft functions than in those with suitable graft function patients (152, 153). In murine allogeneic BM transplant, specific Treg depletion in recipient mice causes a significant reduction in the number of donor hematopoietic cells engrafted (154). As expected, this evidence reinforces the idea that not only CD4^+^ T lymphocytes impact grafting success in allogeneic hematopoietic transplants, but also different CD4+ T lymphocytes exert other influences in hematopoietic niches. Adoptive transfer of Treg cells enhanced donor BM grafting and boosted B cell reconstitution through modulation of IL-7 production by endothelial and stromal cells (155). Additionally, the CD150^high^ CD73^+^ CD39^+^ Treg T lymphocytes subpopulation can maintain hematopoietic stem cell quiescence through adenosine generation, which protects these cells from oxidative stress (156). Using a murine model of allogeneic hematopoietic transplant without immunosuppression, it was demonstrated that Treg cells were able to provide an immune privilege niche responsible for the survival and persistence of allogeneic hematopoietic stem cells in the recipient mice, mediated by IL-10 production. *In vivo* imaging analyses demonstrated that Treg cells are preferentially localized on the endosteal surface of trabecular bone, and these cells were nearby, forming clusters around the allogeneic hematopoietic cells. Reciprocally, the allogeneic hematopoietic cells were lost after Treg lymphocytes were depleted in the host mice (154, 157).

Monteiro JP and colleagues provided substantial evidence that the CD4^+^ T lymphocytes directly influences normal hematopoiesis. T-cell-deficient mice (nu/nu and SCID mice) are granulopenic and there is an accumulation of hematopoietic progenitors in BM due to a maturation arrest of these cells. Curiously, reconstitution of these mice with purified CD4^+^ restores normal levels of mature myeloid peripheral cells and progenitors in BM, which was not observed in animals reconstituted with purified CD8^+^ T lymphocytes. Furthermore, the influence of these CD4^+^ T lymphocytes in the hematopoietic compartment requires recognition of the cognate antigen to activate BM T-helper cells (121).

Experimental approaches using knockout mice have pointed to a differential effect of T-helper subtypes on normal hematopoiesis. STAT4^-^/^-^ mice dramatically impaired differentiation of Th1 cells and displayed a Th2 skewed phenotype. These animals show decreased myeloid progenitor numbers; these cells were slow or non-cycling compared to wild-type mice. Restoring the STAT4 exclusively in T cells was sufficient to recover hematopoietic progenitor cell numbers and activity, and this effect was associated with oncostatin M secreted by Th1 cells (158).

Proportionally, among the CD4^+^ compartment, Foxp3^+^ Treg cells are in high frequency in BM compared to spleen and lymph nodes (124, 154, 159). This population has a pivotal role in maintaining the BM hematopoietic niches. Treg-depleted mice showed alterations in BM hematopoietic populations, with a significative increase in the frequencies of hematopoietic progenitor cells, lymphoid-primed multipotent progenitor, and myeloid cells. In contrast, the B-cell populations (in all stages of maturation) are decreased in these animals compared to the controls. Indeed, in BM transplant experiments with host-Treg depletion, donor engraftment is impaired, with a critical delay in donor B cell reconstitution suggesting a role for T reg in hematopoietic engraftment.

In some tumor types, disseminated tumor cells gain access to the BM, compete, and usurp the niche previously occupied by hematopoietic stem and progenitor cells, taking advantage of this microenvironment and settling in. This complex process requires many steps and disturbs the BM microenvironment (160, 161). In a murine model, the metastatic prostate cancer cells directly compete and lodge in endosteal hematopoietic niches. Moreover, when these animals were submitted for BM transplant, the tumor cells competed and prevented the hematopoietic stem cell engraftment in this site (162). Drawing a parallel with normal hematopoiesis, supposedly, CD4^+^ T lymphocytes also influence and modulate the changes caused by tumor cells in the hematopoietic niches in BM. However, their role in this context remains to be elucidated. By preventing the occupation of hematopoietic niches or even helping with tumor colonization of the bone, CD4^+^ T lymphocytes indeed impact bone metastasis.

Altogether, T cells act physiologically in the bone marrow, balancing hematopoiesis and the bone remodeling system. As such, they use molecules common to both systems. Particularly in pathologies such as cancer and bone metastasis, T lymphocyte action over the bone relies on shared molecules, which are the master regulators of the bone remodeling system. Regarding hematopoiesis, it is not new that T cells secrete molecules that target the hematopoietic compartment, not only in the absence of extreme demand (121). These studies are still not abundant in the literature, and further studies will shed light on the possibility of interfering with bone marrow-born hematologic malignancies as well as metastatic colonization of the bone (**Figure 2**).

#### T cells in bone marrow tumors and bone metastases disrupt tissue remodeling, leading to bone lesions

During pathological conditions, the delicate equilibrium of bone metabolism is disrupted, mostly leading to excessive bone resorption or excess bone formation (163, 164). Cancer-induced bone disease is one of the most common features in oncology patients, being more frequent in patients with myeloma, breast, prostate, renal, melanoma, thyroid, lung, and bladder tumors (165–167). In this breakdown of bone homeostasis, CD4^+^ T lymphocytes act, exacerbating the harmful effects. Indeed, an increase in CD4^+^ T lymphocytes in BM in cancer patients has already been observed, especially in those with metastatic bone disease (124, 168). In the BM of multiple myeloma patients, a significant increase in Th17 lymphocytes and a decrease in Treg and Th1 T cells was observed compared to regular donors. *In vitro* osteoclastogenic assay demonstrated that Th17 from BM myeloma patients was more effective in inducing osteoclast differentiation, suggesting a relationship between Th17 cells and the lytic bone lesions in multiple myeloma (169). In line, high serum concentrations of IL-17A in myeloma patients are associated with severe bone osteolytic disease and higher fracture incidence than those with lower IL-17A concentrations (170).

Bone osteoblastic lesions are the lethal and significant clinical problem consequences of metastatic prostate cancer cells in this microenvironment (171). Along with the evidence that the metastatic prostate tumor cells, located preferentially in an osteoblastic niche, can modulate the osteoblasts phenotype and activity in their favor, and CD4^+^ T lymphocytes present in bone could also be involved in this phenomenon (171–173). It has already been observed that Treg cells were significantly higher in BM from patients with prostate cancer bone metastasis than in those without bone involvement (174). *In vitro* assay has shown that Treg could suppress osteoclast differentiation and function and stimulate osteoblasts proliferation and differentiation (131, 174, 175). These data indicate that Treg may be implicated in suppressing osteoclastogenesis and stimulating osteoblasts, generating osteoblastic lesions frequently observed in prostate cancer bone metastasis.

In a metastatic breast cancer murine model, we showed that tumor-primed Th17 RANKL^+^ lymphocytes are crucial for preparing the pre-metastatic bone marrow niche. The RANKL, derived from anti-tumor T cells, induces osteoclastogenesis, leading to an increase in bone resorption before the arrival of metastatic cells, generating a rich and ideal microenvironment for establishing these cells as a necessary pre-metastatic niche. Also, the transfer of just these cells to the athymic hosts induces a pre-metastatic osteolytic disease, which depends on RANKL expression by these lymphocytes (**Figure 2**) (84, 146).

### Maintenance of the skin barrier depends on the balance of the skin’s resident T lymphocytes

#### The specific segregation of APCs in the skin layers is essential for maintaining the balance of T cell phenotypes

The skin is the largest organ in the human body, which, like some mucosal surfaces such as the intestines and lungs, acts as a physical barrier, protecting the body against invading agents. This protection is achieved through the histological organization of the skin. The skin can be divided into the epidermis, dermis, and hypodermis. The epidermis can be subdivided into the *stratum corneum*, *granulosum*, *stratum spinosum*, and *stratum basale*. Immune system cells, such as specific APC and subtypes of T lymphocytes, populate these histological layers of the skin in a differentiated and highly organized manner. Human skin, which covers about 18000 cm^2^, contains around 1 million T cells per cm^2^, totaling almost 20 billion T cells. Most of the skin’s resident T cells are CD4 effector memory cells that express CLA and CCR4, with only a tiny fraction of central memory cells that express CCR7 and CD62L (176). The TCR repertoire presented is varied, as is found in cells circulating in the blood. However, there is an accumulation of cells with TCR that have Vβ7, Vβ14, and Vβ16, suggesting that the cells that make up the memory pool do not come from random sampling. Still, they are probably selected by self-antigens, microflora, or infections (177).

In mice, at steady-state, the epidermis is populated by resident dendritic epidermal T cells (DETC^-^CD4^-^CD8^-^Thy-1^+^) but not CD4^+^ or CD8^+^ T cells, which infiltrate the tissue upon infections. DETC express TCRγδ and have a restricted repertoire. They play a crucial role in skin homeostasis and wound healing through the expression of IGF-1 (178–180). In contrast, the human epidermis is populated by both γδ (18% of T cells) and ɑβ T lymphocytes (82% of T cells). Both populations produce IGF-1, acting directly in maintaining skin integrity and wound healing, with peak production 24 hours after the injury (181, 182). Regarding CD4 and CD8 T cells, CD4^+^ T lymphocytes account for most of them (72%). Despite having more CD4^+^ than CD8^+^ T lymphocytes in the epidermis, the epidermis still has more CD8^+^ T lymphocytes than the dermis (183).

Atopic dermatitis (AD), or eczema, is the most common chronic inflammatory disease of the skin. It is characterized by malfunctioning of the skin barrier with dysbiosis of the skin microflora (184). Although the etiology of AD is not entirely understood, the premise that skin barrier malfunction is a cause and not a consequence of AD is widely accepted due to its high prevalence in individuals with a filaggrin mutation and the fact that even resolving the inflammation, the barrier is not re-established (185, 186). Along with these skin barrier problems, dysbiosis can occur, progressing to colonization of *Staphylococcus aureus*, which can increase the severity of the disease (187, 188). With the stratum corneum barrier compromised, the location of the LC is advantageous for the recognition and phagocytosis of *S aureus*. In turn, Langerhans cells (LC) activate Th17 cells and Tγδ lymphocytes that express IL-17A and IL-17F, which would be necessary to respond and eliminate this bacterium (189). On the other hand, IL-17A induces a reduction in the expression of filaggrin and loricrin by keratinocytes, which in turn impairs the integrity of the skin barrier, considering that the former is involved in the aggregation of keratin 1 and 10 in the cornified envelope. The second is highly insoluble and hydrophobic and is essential in reinforcing the cornified envelope (190, 191). Besides that, lipoteichoic acid (LTA), from the wall of invading *S. aureus*, has a role inducing Th2 response, which, in turn, downregulates the expression of important proteins in maintaining the integrity of the skin barrier such as filaggrin, ceramide, involucrin, loricrin and desmoglein 3 (192–196). Reduction in the expression of these molecules is accompanied by the shift from a Th2 response to a Th1 response, more commonly found in chronic AD (197). High concentrations of IFN-γ interfere with melanocyte maturation, altering the skin barrier’s homeostasis. This alteration in the skin barrier can be even more severe if in the presence of high concentrations of IL-17 and TNF-α, in addition to IFN-γ (198, 199). Candida albicans infection is also an infection of clinical importance in individuals with AD. Due to the loss of epidermal barrier integrity and dysbiosis, *C.* albicans infection can occur in the skin, and LC can recognize their β (1, 3)-glucans through dectin-1, a pattern recognition receptor. Activation through dectin-1 induces the production of IL-6 by the LC, which is important for the differentiation of Th cells into the Th17 subtype, critical for fungi elimination, but again interfering in skin barrier downregulating the expression of filaggrin and loricrin (200–202).

**Figure 3:**
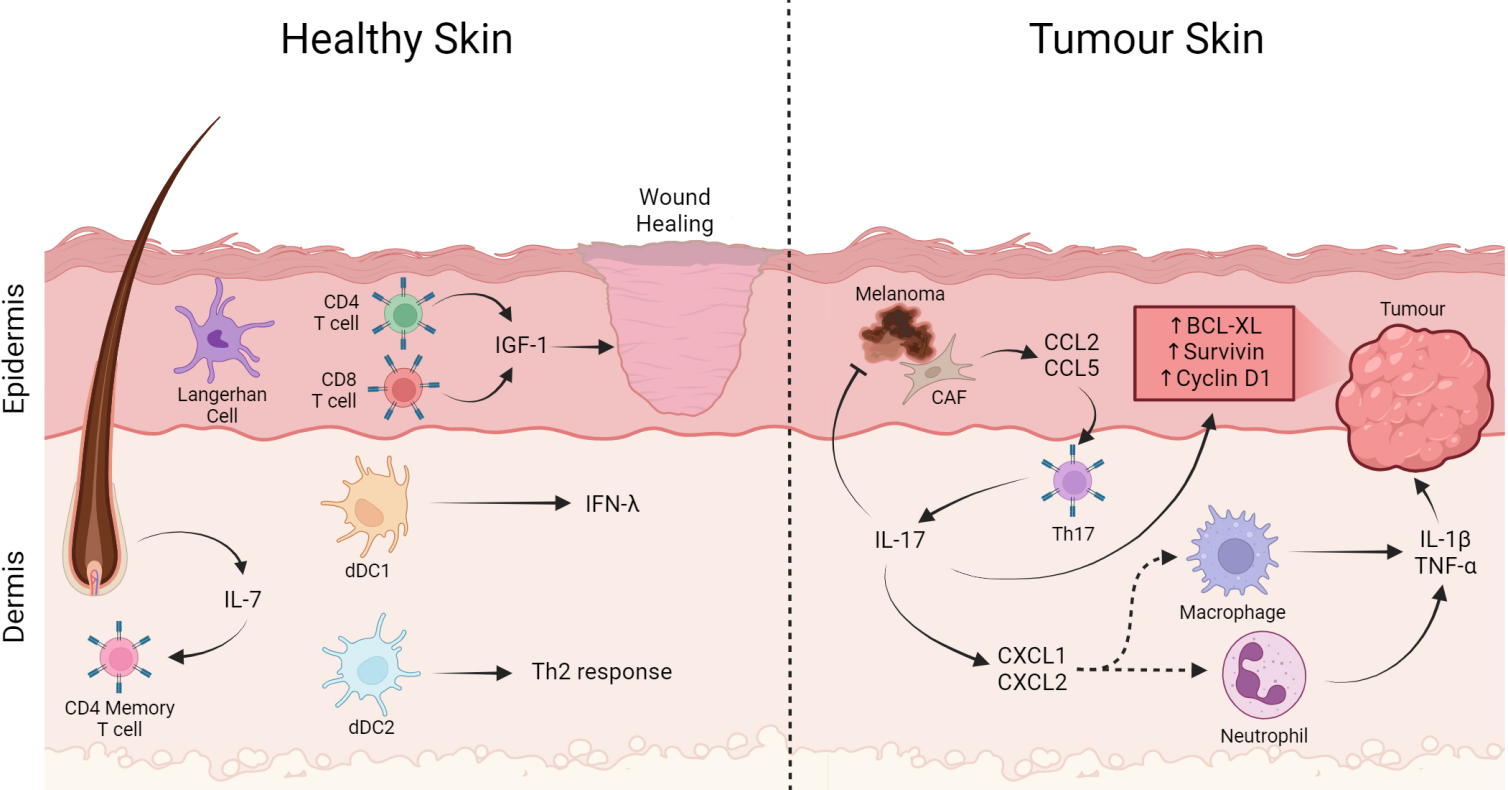
T cells in the skin play a role in wound healing and tumor pathogenesis. In healthy skin, Langerhan cells are the most abundant APC, besides that CD4^+^ and CD8^+^ T lymphocytes cells have an important role in wound healing producing IGF-1. On the other hand, memory T cells are in the dermis close to the follicle, an important producer of IL-7 that helps maintain the memory profile. Furthermore, dDC1 producing IFN-λ capable of cross-presentation and dDC2 important for Th2 generation. On the other hand, in tumor-affected skin Th17 cells expressing CXCL1 and CXCL2 contribute to the recruitment of macrophages and neutrophils producing IL-1β and TNF-α. Th17 directly through IL17 production induce expression of anti-apoptotic proteins and carcinogenic promoters such as Bcl-XL, Survivin, and Cyclin D1. Furthermore, cancer associated fibroblast cells produce CCL2 and CCL5, which are important for the recruitment of Th17 cells, contributing to the increase in this cell in the infiltrate. Created with BioRender.com.

Although LC is the only antigen-presenting cells in the epidermis, the dermis has others, such as type I (dDC1 or dermal cDC1, called CD141^+^ DC in humans) and type II dermal dendritic cells (dDC2, or dermal cDC2, called CD1c^+^ DC in humans) (203–206). Dermal cDC1 is the minority dendritic cell population of the dermis (207). This minority population is the most significant producer of IFN-λ after poly-IC inoculation and can cross-present antigens (208), which is essential in tumor response. Furthermore, in a *Leishmania major* infection model, in which the Th1 response is necessary for the resolution of the infection, mice deficient in BATF3 (DC1 deficient) demonstrated an absence of parasite control response due to non-expression of IL-12 by dDC1, essential for differentiation to Th1 (209). On the other hand, dermal cDC2 is the most abundant DC in the dermis (Henri et al. - 2010). Mice that do not have dermal cDC2 cannot mount an antigen-specific Th2 response in an immunization protocol nor in the hookworm infection *Nippostrongylus braziliensis* (210). This is confirmed through a contact hypersensitivity model by dermal transfer of cDC2 from a FITC-immunized mouse (211). Furthermore, immunization with OVA and TSLP typically induces a robust Th2-type response. However, mice with DC deficiency in STAT5 cannot mount a Th2 response but can mount a Th1 type. The same is observed in an immunization model by FITC combined with dibutyl phthalate (212). These observations suggest a crucial role for TSLP-induced STAT5 signaling in the induction of the Th2 response by dermal cDC2. Most dermic T lymphocytes are close to skin appendages such as glands and hair follicles. Hair follicle keratinocytes express IL-7, crucial for CD4^+^ T memory lymphocytes maintenance (213). In addition to memory T cells, Treg lymphocytes also populate the dermis near hair follicles. These Tregs have a more immunoregulatory profile than the Tregs found in draining lymph nodes and make the site immune-privileged and vital in protecting against alopecia (214). In the dermis, as in the epidermis, most T cells are CD4^+^ (85%). Although most T cells are resident, about 15%-25% of T cells are recirculating (183). Most skin CD4^+^ T effector memory lymphocytes are polarized to the Th1 profile, while approximately 7% are Th2 polarized, and 5% are Treg. These Tregs are functional and can inhibit the proliferation of effector T lymphocytes in a contact-dependent manner but independent of IL-10 and TGF-β. Furthermore, they can expand independently of antigen through IL-15 signaling and contact with dermal fibroblasts, as most memory T cells do (**Figure 3**) (176, 215).

#### Th17 cell-mediated inflammation potentiates tumorigenesis in the skin

This specific distribution of APCs in the epidermal and dermal layers and the T-response findings suggest a layer-specific response organization. Indeed, psoriasis is an example of response dysregulation culminating in a skin disease. Psoriasis is a skin disease in which plasmacytoid DC (pDC) are believed to produce type-I interferon triggered by virus recognition. This type-I interferon activates DC and produces TNF-α, IL-12, and IL-23, leading to Th1/Th17 cell differentiation and unbalancing tissue immunity (216–218). The same seems to play a role in tumor development in the skin, considering that inflammation is one of the critical processes in non-melanoma skin carcinogenesis (219). Wang and cols. showed, using the classic murine model of skin carcinogenesis through the administration of 12-dimenthylbenz[a] anthracene (DMBA) and 12-o-tetradecanoylphorbol-13-acetate (TPA) in IL17^-/-^ mice, that the production of IL-17 by CD4^+^ T lymphocytes induces the production of CXCL1, CXCL2 and the infiltration of neutrophils and macrophages with increased levels of TNF-α and IL-1β. Furthermore, IL-17 production by CD4^+^ T lymphocytes is required to induce the expression of anti-apoptotic proteins and carcinogenesis promoters such as Bcl-XL, Survivin, and Cyclin D1 that can promote epithelial proliferation (220, 221). In addition, individuals with squamous or basal cell carcinoma (BCC) have higher serum IL-17 levels than healthy individuals, suggesting that this phenomenon may also occur in humans (222). In a study with 39 patients with BCC, Pellegrini and colleagues showed that the number of IL-17-producing CD4^+^ T lymphocytes correlated positively with the severity of the inflammatory infiltrate in the tumor (223). Melanoma is a skin cancer with the worst prognosis and high rates of distant disease (224). The role of the immune system in eliminating melanoma becomes apparent with the success of immunotherapies such as immune checkpoint inhibitors (225). However, the immune system’s role in promoting melanoma is unclear. In melanoma, cancer-associated fibroblasts (CAF) produce CCL2 and CCL5 that attract Th17 cells that, in turn, can infiltrate and accumulate in the tumor (226). In mice, the IL-17 production inhibited in the tumor environment developed smaller melanomas and lower levels of MMP9, VEGF, and CD31, suggesting less angiogenesis in the TME (227). This was also demonstrated using an IL-17 knockout animal model, in which the production of VEGF and MMP9 induced by IL-17 depended on the IL6/Stat3 pathway (221). On the other hand, Th17 cells expressing IFN-γ mediate melanoma eradication in an *in vivo* murine model using the B16 cell line (228). Furthermore, the inflammation promoted by Th17 enhances the presentation of tumor antigens by CD8α^+^ DC to CD8 cells, thus enhancing the anti-tumor response (229).

So, in physiology, T cells are critical to maintain epithelial integrity. However, continuous antigen stimulation and T-cell activity can act as tumor promoters, disrupting the balance of epithelial cell renewal (**Figure 3**).

### T cells in the liver: from hyporesponsive to Th1-mediated protection

#### The liver displays an environment with adaptive immune hyporesponsiveness

The liver is an immunologically hyporesponsive organ, and the hepatic microenvironment seems to impose the immunosuppressive profile (230). The liver is the second most frequent target organ of distant disease, perhaps due to its immunosuppressive profile (231, 232). In apparent contradiction with this fact, the liver has an essential role in the acute-phase response of inflammation, producing mediators like C reactive protein (233) and Mannose-Binding Lectin (234), which act in the immediate innate immune response. In addition, it harbors many resident immune cells, from innate to adaptive immune responses, like Natural Killer (235), invariant Natural Killer T cells (236), and Kupffer cells (237).

Liver sinusoidal endothelial cells (LSEC) constitutively express IL-10 and PD-L1, which could be, in fact, important to suppress the immune response against gut-derived peptides arriving through the portal vein (238, 239). Conversely, LESC via integrin α4 and vascular adhesion protein (VAP)-1 recruit Th1 and Th2, respectively (230). Besides that, during a viral liver infection, activation of CD4 and CD8 cells is essential to control viral titers and the disease. So, resident T cells must bypass the suppressive environment while keeping the organ homeostasis balanced.

T cells access the liver from the portal vein and the hepatic artery. So, through this unique circulatory supply, T cells compose part of the organ’s adaptive immunity cells. Although T cell activation does not frequently occur in the liver, they are essential in the response against pathogens (240). Once the infection is resolved, they remain in the tissue as memory cells (as in other tissues). Classic αβ T lymphocytes can represent up to 50% of the lymphocytes in the liver, and different from what occurs in the blood, there is a lower frequency of CD4^+^ and a higher frequency of CD8^+^ T lymphocytes (241). Liver resident memory CD8^+^ T lymphocytes are very well studied; they express CD69, CD103 (242) and low levels of CCR7, CD62L, and S1PR1 (243), essential molecules for tissue exit. Still, they also express homing molecules that are not liver-specific, such as CXCR3 and CXCR6 (244). In addition to the rapid response to reinfection (245), Koda and colleagues demonstrated that in High-fat and high-cholesterol (HFHC), diet-induced model of Non-alcoholic steatohepatitis (NASH), resident memory CD8^+^ T lymphocytes are maintained by the cytokine IL-15 locally produced, and induce apoptosis of hepatic stellate cells (HSCs) via Fas-FasL, restoring tissue homeostasis and preventing HSC-mediated fibrosis (246). So, when tissue homeostasis is interrupted, resident T cells are triggered to restore the balance within the microenvironment.

In terms of homeostasis, liver regeneration models have been studied to understand tissue homeostasis processes. Liver regeneration involves cell proliferation and differentiation, and cytokines play a key role in this process. HSCs produce Hepatocyte Growth Factor (HGF) and act on hepatocytes, promoting their proliferation and survival (247). HSC also produces TGF-β and plays a role in tissue remodeling and the formation of new blood vessels, which are essential for liver regeneration (248). Kupffer cells produce TNF-α and IL-6 in response to tissue injury. These cytokines act on the proliferation and survival of hepatocytes (249, 250). IL-6 acts on hepatocytes by activating the JAK/STAT signaling pathway (250). Activated T cells can produce all the mentioned cytokines under different stimuli. This suggests that liver resident T cells contribute to liver homeostasis by helping regulate cell growth and differentiation in homeostatic situations.

**Figure 4:**
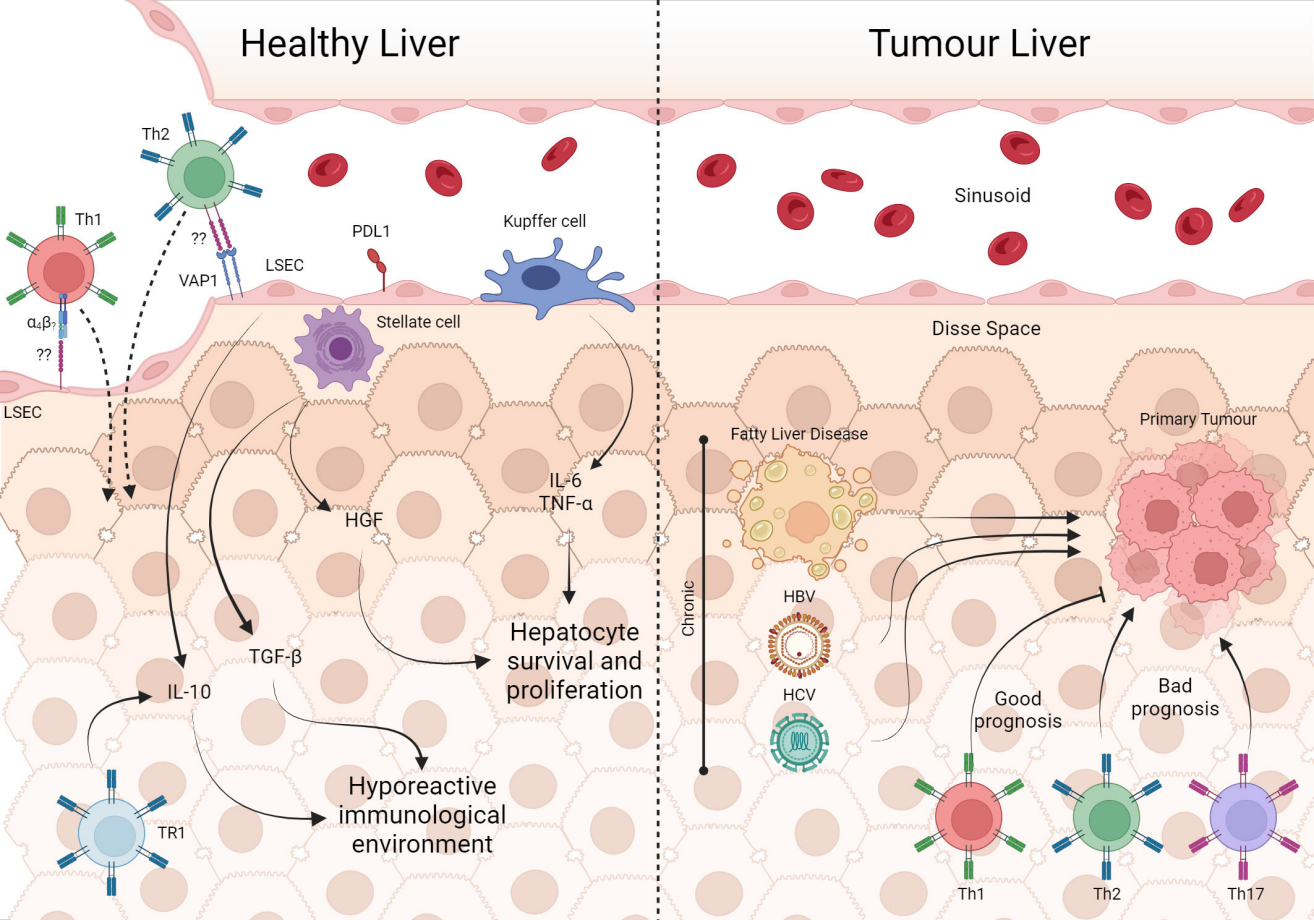
The involvement of T cells in healthy liver and tumor processes. In the unique architecture of the liver, Kupffer cells, through IL-6 e TNF-α, are involved in the survival and proliferation of hepatocytes, as well as HGF from stellate cells. Furthermore, the production of IL-10 by TR1 and LSEC in addition to TGF-β from stellate cells maintains the hepatic microenvironment hyporesponsive. However, LSEC can recruit Th2 and Th1 via VAP-1 and α4 integrin, respectively which are important in infection contexts. Fatty liver disease, HBV and HCV are some of the risk factors for the development of liver tumors. With tumor establishment, cells from the Th1, Th2 and Th17 profiles are present in the tissue, with Th1 being associated with a good prognosis of the disease. Created with BioRender.com.

As mentioned, the liver has a higher percentage of CD8 than of CD4^+^ T lymphocytes, and there are epidemiologically critical viral infections in the organ, such as Hepatitis B virus (HBV) and hepatitis C virus (HCV) infections, which implies a more significant amount of information regarding CD8^+^ T lymphocytes in the liver. However, the importance of CD4^+^ T lymphocytes in restoring local homeostasis is remarkable. It has already been shown that CD4^+^ T lymphocytes are present in healthy individuals’ perfused liver; these cells reside in the organ and probably have a memory profile (251). Indeed, data from mice liver T cells (αβ T cells and NK T cells) under normal conditions have shown that these cells are IFN-γ producers (252). In some way, this hyporeactive immunological tissue is more receptive to apparently activated cells. However, the outcome of these cells when penetrating the tissue is not known. Perhaps it is the control of the production of inflammatory cytokines at levels that do not cause tissue injury. Indeed, the liver appears to control the effector response. In a model of induction of oral tolerance to ovalbumin in rats with portocaval shunt, a surgery that promotes the connection of the portal vein with the inferior vena cava, diverting the blood flow that would pass through the liver, showed that these animals presented a reduction in the tolerance measured by DTH, suggesting that the hepatic microenvironment induces control of the response to food antigens (253). This also occurs in a load of microbial products from the intestinal microbiota that reaches the liver via the portal vein. The importance of T cells in immune tolerance is already known, especially when it comes to Treg cells, and although we have thymic Treg cells, locally induced Treg cells are essential for tolerance to non-self antigens, such as antigens acquired through the oral route (254). Non-conventional Treg cells (FOXP3^-^), such as Type 1 regulatory T cells (Tr1^-^CD4^+^FOXP3^-)^, are also crucial in generating local tolerance, commonly found in mucosa-associated lymphoid tissue (MALT), also affecting inhibition of the response through the production of the cytokine IL-10 (**Figure 4**) (255, 256).

#### Th1 response is protective against hepatitis and liver tumors

When there is aggression, such as HBV and HCV infections, the local equilibrium will be disrupted, and an effective antiviral Th1 immune response, probably primed outside the organ, occurs transitorily. The class switch on T cell function inside the tissue, implying secretion of molecules not part of the organ remodeling system or inadequate production of cytokines involved in tissue maintenance will surely damage the tissue. So, despite its hyporeactive state, necessary for establishing tolerance, the tissue can also mount a response against tumors and pathogens, even when the infection often becomes chronic. In HCV infection, Th1 cells produce IL-2, culminating in CD4^+^ and CD8^+^ T lymphocytes expansion. The absence of the Th1 response leads to an inefficient response that evolves into a chronic infection (257, 258). An analysis of PBMC from patients with HCV revealed that specific CD4^+^ T lymphocytes indeed respond to stimulation with viral proteins and induce IFN-γ, TNF-α, and IL-2, the classic Th1 response expected in viral infections. However, some cells recognize viral proteins but do not increase after encountering cognate peptides and express more PD-1 and CTLA-4 than HCV-specific cells that can proliferate in response to viral proteins (259). The passage through the hepatic microenvironment probably suppresses the extensive antiviral response. Chronic HCV-infected patients have a lower percentage of circulating virus-specific CD4^+^ T lymphocytes. In contrast, individuals who have resolved the infection (tested HCV RNA negative) have stable levels of virus-specific CD4^+^ T lymphocytes, even 100 days after infection (260). The response pattern appears to be the same in HBV. As the frequency of virus-specific CD4^+^ T lymphocytes is low, generating the expansion of virus-responsive cells *in vitro* is necessary. These assays demonstrated that in HBV infection, patients negative for viral antigens present greater dominance of cells IFN-γ-producing cells compared to individuals who test positive for viral antigens. The same did not occur with the TNF-α-producing CD4^+^ T lymphocytes; even though TNF-α is necessary to resolve the infection, this cytokine is related to liver damage. In this context, we observe cytokines produced by CD4^+^ T lymphocytes as possible defining factors for the outcome of the disease (261). Although the pathogen dictates the Th1 response profile, resident cells establish themselves as memory cells after clearing infection, which will be maintained locally by either cytokine signaling or MHC/peptide stimulation. As resident cells do not harm the tissue and T cells have the potential to produce all the major cytokines involved in tissue regeneration, we propose that they are integrated into liver remodeling mechanisms.

The establishment of a chronic inflammatory state is associated with the appearance of primary liver cancer. Therefore, infections with HBV and HCV viruses, alcoholic liver disease, and fatty liver disease are the main risk factors for the development of hepatocellular carcinoma (HCC), making the liver the sixth most frequent primary tumor organ (262). There are two main types of liver cancer: hepatocellular carcinoma (HCC), which comprises 80–90% of primary liver cancers, and cholangiocarcinoma (CCA), which accounts for 10–15% of the cases (263). During the development of the different types of primary diseases that lead to tumor formation in the liver, innate immunity is involved, and several profiles of T cells are affected. HCC has a lymphocytic tumoral infiltrate (TIL) characterized by heterogeneity (264). However, each phenotype has a different clinical outcome. The establishment of a Th17 profile appears to correlate with poor prognosis, so TILs with a higher density of IL17 and its receptor are associated with lower survival (69). On the other hand, the presence of Th1 cells is associated with a good prognosis (265). Despite not addressing the cytokines produced by these cells, the study also pointed out that the higher expression of Th2 genes is associated with poor prognosis. This cell phenotype is more present in patients in stage IV (265). Moreover, the CD4^+^ T lymphocytes phenotype response also appears to be involved in metastatic establishment in primary liver tumors. A study that compared samples of tumors from patients with HCC with and without metastasis showed that tumors that metastasized showed a phenotypic change in the T response from Th1 to Th2, with increased cytokines such as IL4 and IL10. Nevertheless, tumors without metastasis seem to maintain a balance between the two types of response (266). Animal models are crucial to understanding the role of CD4 LTs in cancer pathology. However, articles that use animal models of liver tumors focus on the role of CD8 LTs, leaving a gap regarding the involvement of CD4^+^ T lymphocytes in tumor development/elimination, and do not consider the role of CD4^+^ T lymphocytes in homeostatic maintenance of the organ (**Figure 4**).

### T cell dynamics and intestinal heterogeneity: balancing inflammation and tolerance in gut homeostasis

#### With its peculiar relationship with the microbiota, the intestine maintains a balance between the Th17/Treg phenotypes

The vast extension of the intestine is marked by tissue heterogeneity related to its role in nutrient absorption. The amount of soluble food antigens in the lumen decreases from the duodenum to the colon; in contrast, the density and variety of commensal bacteria increase from the duodenum to the colon (267). In fact, there is a two-way modulating system between commensal bacteria and the IS.

In this way, bacteria like *Lactobacillus reuteri* and *Bifidobacterium animalis* induce CD4^+^ T lymphocytes differentiation towards the Th17 profile, producing IL-17 (A-F) and IL-22 (268, 269). The recognition of intestinal microbiota antigens by Th17 cells is important in situations of intestinal barrier breakdown (270). A study by Ivanov and colleagues demonstrated the crucial role of Th17 cells and their signature cytokine, IL-17, in protecting against *Citrobacter rodentium* infection in the intestine (26). Data suggest that cytokines produced by this cell subtype, such as IL-17A, may be associated with the induction of antimicrobial peptide production by epithelial cells (271), and Th17 cells can recruit and activate neutrophils, further enhancing the immune response against invading pathogens (39). The balance in the response of Th17 cells and neutrophils is essential in the control of intestinal homeostasis as it controls infections and, at the same time, avoids exacerbated tissue damage generated by the local response; dysregulation of this interplay can lead to chronic intestinal inflammation, as observed in conditions such as inflammatory bowel disease (IBD) and cancer.

In addition to the classic production of the cytokine IL-17, Th17 cells can also produce cytokines such as IL-22. The receptor complex of IL-22 (composed of L-22R1 and IL-10R2) is primarily expressed on epithelial cells in the intestine. Upon activation, IL-22 signaling triggers a cascade of events contributing to tissue repair and protection. IL-22 also promotes the production of antimicrobial peptides (AMPs) by intestinal epithelial cells. AMPs, such as defensins and regenerating islet-derived protein three gamma (RegIIIγ), have potent antimicrobial properties and contribute to maintaining intestinal barrier function by preventing the invasion and colonization of pathogens (272). Furthermore, IL-22 can promote epithelial cell proliferation and survival and stimulate extracellular matrix (ECM) production, thereby supporting tissue remodeling and repair (273).

So, IL-17 recruits polymorphonuclear cells in gut homeostasis and activates local inflammation, while IL-22 acts on epithelial regeneration. Th17 cells are differentiated and expanded under the influence of IL-6/IL-23/TGFβ, meaning a balance between what we didactically name inflammation and anti-inflammation represented by the cytokines IL-6/IL-23 and TGFβ, respectively. However, when T cells are activated, depending on how it happens, it will produce IL-2, a master mitogenic cytokine. With IL-2 concentrations rising and TGFβ production ongoing, T cells can become Treg cells. Foxp3+ Treg cells have been recognized as potent suppressors of T-cell responses. Most gut Treg is generated at the periphery, so they differentiate locally in response to antigens from microbiota under the influence of this environment rich in TGFβ. Gut Treg cells seem to have a direct role in tissue physiology, acting on the renewal of epithelial stem cells by producing IL-10 (274). In addition to conventional Treg cells, the intestine has also described a Treg subpopulation that co-expresses RORγt and Helios-Nrp1-Foxp3+. These cells have been attributed to suppressing the immune response in some models of inflammation due to high levels of IL-10 expression, even though they have lower levels of CTLA4 expression than T reg FOXP3+RORγt- (269, 275). Lin and colleagues recently described that intestinal murine Treg (CD83+CD62LLow) also produces IL-27, essential for controlling the local Th17 inflammatory response (276).

**Figure 5:**
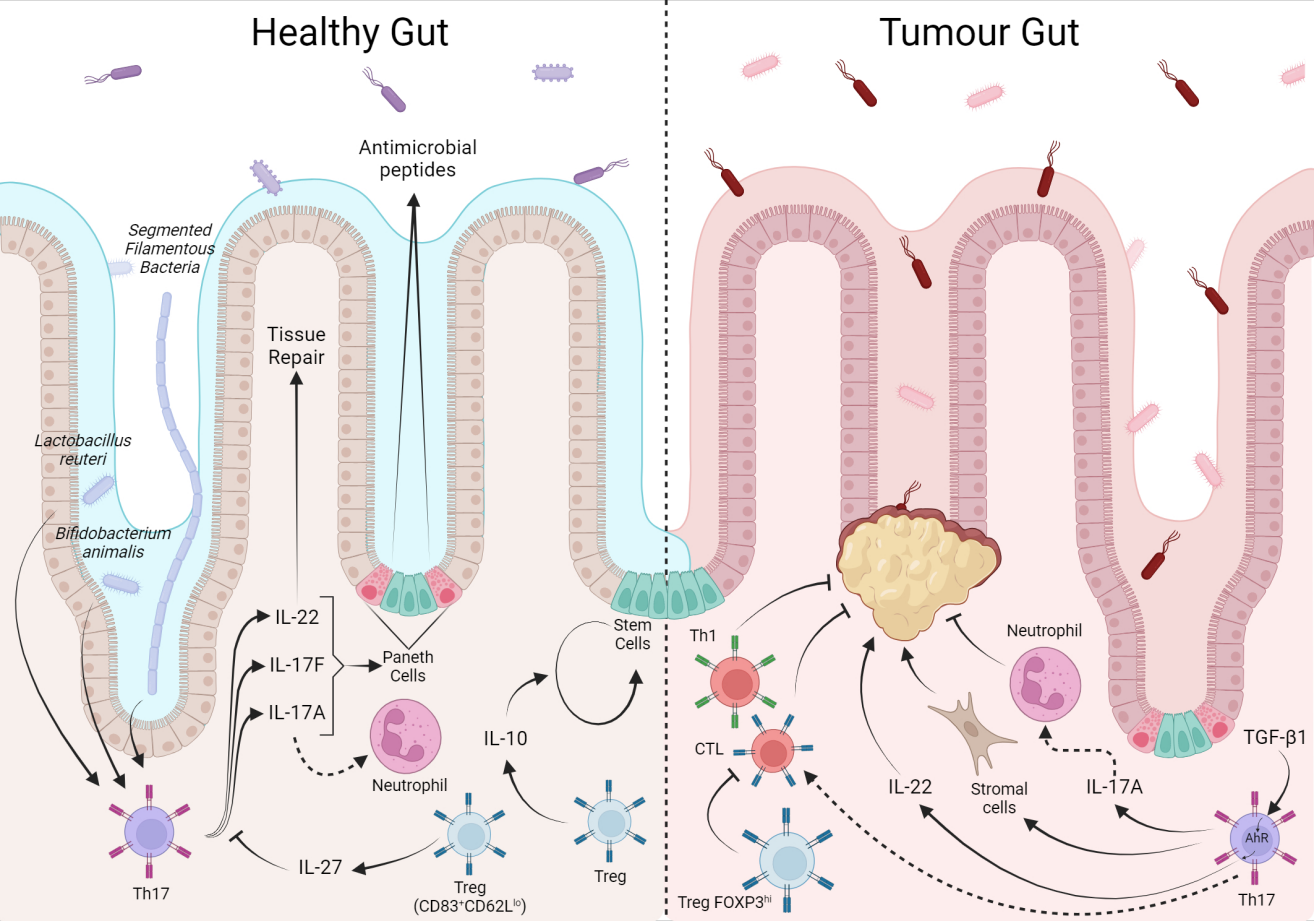
The T cell response in the intestine is balanced between tolerance to local microbiota and food antigens, but has a prognostic factor when in response to colorectal cancer. The local microbiota modulates the T cell phenotype, *Lactobacillus reuteri* and *Bifidobacterium animalis* induce the Th17 phenotype whose production of IL-22, 1L-17A/F, act in tissue repair, and induction of antimicrobial peptides production. IL-10 and IL-27 from Treg assist in tissue self-renewal and control of the local T response, respectively. In CRC, Th17 cells through IL17-A recruit antimoral neutrophils while they can also stimulate stromal cells in favor of tumor. Furthermore, under TGF-β1 stimulation from the tumor environment, IL-22 favors tumor growth. In contrast, local Treg inhibit tumor-inhibiting CD8^+^ and Th1.Created with BioRender.com.

The intestine is a heterogeneous organ, and each intestinal portion has its function and network of draining LNs. It makes sense that the T cells in LN, which drain different portions of the intestine, are phenotypically distinct and in harmony with the segment function. In this sense, Esterházy and colleagues demonstrated that the monocolonization with Segmented Filamentous Bacteria led to a Th17 cell differentiation with a lower frequency in the Duodenum and a higher frequency in the Cecum. In contrast, immunization with OVA by gavage led to a high Treg frequency in the Duodenum and a lower frequency in the Cecum. These data suggest a potentially pro-inflammatory profile in the more distal intestinal portions, with a higher concentration of commensal bacteria, while preserving the T-cell profile throughout the entire organ (13). Altogether, the intestines show us that the immune system indeed has a patrolling rule, but this is in harmony with keeping health and respecting the local functionalities. It cooperates with the local tissue, participating in the epithelial reconstitution. It enables our feeding by making us tolerant to food antigens where food antigens are mostly absorbed (small intestine) and fight off undesirable intruders where this risk is higher (colon) (**Figure 5**).

#### T lymphocytes in colorectal cancer are linked to a good prognosis, but the connection between infiltration quality and disease severity involves different characteristics

Unlike the other organs mentioned in this review, there is a well-established immunoscore correlating T cells with prognosis in colorectal cancer (CRC). Galon and colleagues conducted one of the pioneering investigations in this area, using primary tumor samples from individuals diagnosed with CRC. The study revealed that patients experiencing tumor recurrence exhibited a decreased count of total T lymphocytes both within the tumor core and in the neighboring tissue. In contrast, those without tumor recurrence displayed an elevated T cell count. Stratifying the samples based on CD3^+^ cell density indicated a clear distinction in disease staging. Patients with heightened CD3 density were associated with stages I, II, and III and demonstrated improved disease-free survival. Conversely, patients with a lower lymphocyte percentage at stage IV experienced diminished disease-free survival. This emphasizes the connection between infiltration and a favorable prognosis, emphasizing the enhanced prognostic significance of tumor-infiltrating lymphocytes (TIL) compared to conventional measures. Moreover, the study highlights the notable influence of the adaptive immune system on tumor dynamics. Additionally, they found a direct association between the existence of markers indicative of Th1 polarization, as well as cytotoxic and memory T cells, and a reduced occurrence of tumor recurrence (277).

Also, a significant amount of infiltrating FOXP3^+^ Treg cells in tumor tissue has already been observed in CRC. Nevertheless, their role remains controversial, with evidence pointing to favorable clinical prognosis and improved survival (78, 278, 279) and associated with poor prognosis (278, 280, 281). Recently, a more detailed investigation regarding the different subsets of Treg cell populations might clarify this contradiction. Saito and colleagues suggested that the functionally distinct subpopulations of tumor-infiltrating Treg cells displayed opposing roles in CRC. Analysis of Treg cells in the CRC tumor site demonstrated two main phenotypes: CD4^+^FOXP3^hi^CD45RA^−^ and CD4^+^FOXP3^lo^CD45RA^−^. Treg with high FOXP3 expression showed suppressive activity, while Treg cells with low FOXP3 expression showed no suppressive activity but secreted proinflammatory cytokines contributing to the antitumor response (282). Although they did not predict clinical outcomes, Th17 were significantly higher in CRC tumor areas than in corresponding healthy tissues (226, 283, 284). *Ex vivo* analysis demonstrated that these cells, besides IL-17, produced TNF-α, IL-21, IL-22, GM-CSF, and IL-8, indicating that CRC-infiltrating Th17 are polyfunctional effector cells. Furthermore, with *in vitro* experimental approaches, CRC-Th17 appears to mediate protumorigenic effects mainly by acting on tumor stroma cells. On the other hand, they promote the recruitment of neutrophils and CD8^+^ T lymphocytes associated with an antitumor response (283). Besides, the amount of Th17 lymphocytes in CRC tissue was associated with the disease stage and was higher in patients in advanced stages than in the early stages (284), possibly indicating different roles during CRC progression.

In the murine model, a significant accumulation of IL-17^+^ Tregs lymphocytes was observed in CRC tumor tissue. Those cells display a different cytokine profile than conventional Treg cells, expressing high levels of proinflammatory cytokines such as IL-17, IL-2, IL-6, TNF-ɑ, and IFN-γ, and no expression of IL-10 but maintaining the immunosuppressive capacity (285–288). Unexpectedly, in an *ex vivo* coculture assay, Ma and Dong demonstrated that the impairment in proliferation of CD8^+^ T in the presence of CRC IL17^+^ Tregs was abolished when IL-17 was blocked, suggesting that the IL-17 cytokine was crucial to this effect (286). Still, in relation to Th17 cells in CRC in an animal model, when the tumor was induced by azoxymethane with dextran sulfate sodium, TGF-β present in the TME induced the differentiation of IL-22-producing Th17 through the expression of aryl hydrocarbon receptor (AHR) contributing to tumor growth. The tumor tended to shrink in TGF receptor knockout animals on T cells, suggesting direct tumor involvement of CD4^+^ T lymphocytes via IL-22 (289). Additionally, in the same model, it was demonstrated that IL-17RD, a receptor of the IL-17 family, has an antitumor effect, showing that the involvement of cytokines produced by Th17 in CRC tumors is divergent even within the same model (290).

Although the presence of infiltrating T cells in CRC has a direct relationship with the prognosis of the disease, it was impossible to establish a relationship between a single type of infiltrate and the prognosis of the disease. The colon microbiota depends on several factors that vary individually, such as the environment, use of medications, and diet. As the microbiota is an essential definer of the local adaptive system, it is suggested that individuals may have certain variations in the phenotype of tissue-specific T cells. Thus, the tumor that adapts to the environment can also present TILs with different phenotypes that can be pro or antitumor (**Figure 5**).

### In the breast tumor animal model, tumor progression involves changes in the phenotype of tissue-specific CD4^+^ T lymphocytes

**Figure 6:**
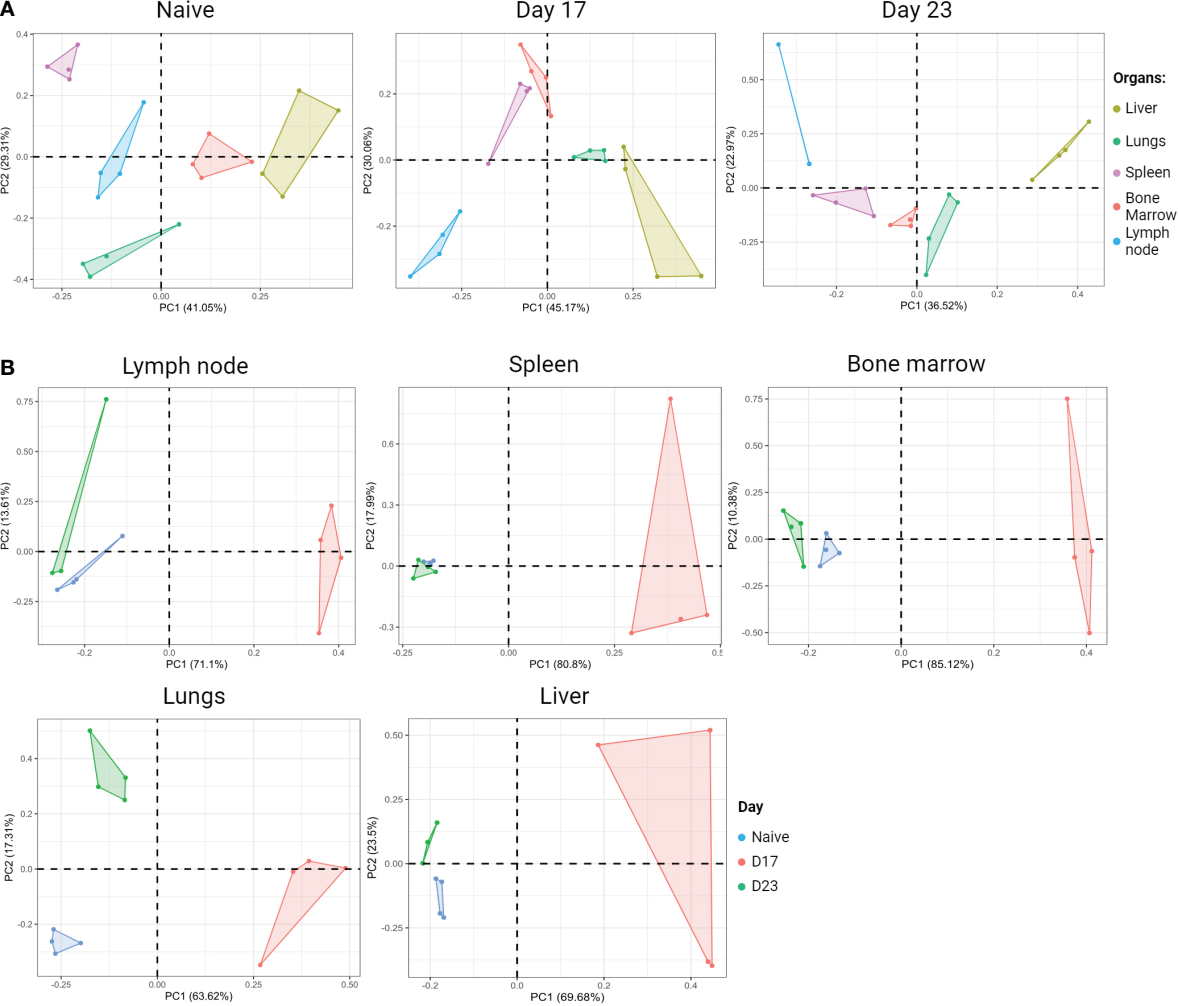
Organ-specific signature based on the cytokine profile of CD4^+^ T cells. BALB/c female mice receive 5x10^4^ 4T1 cells inoculated subcutaneously in the fourth right mammary pad. On the days shown, animals were injected IV with anti-Thy1.2 mAb and three minutes later were euthanized. Cell suspension was obtained from each organ and stained for flow cytometry for CD4, TNF-α, IFN-y, IL-13, IL-17A, IL17-F and GM-CSF. Thy1.2^+^ cells were excluded from the analysis and the results shown were from gated CD4^+^ T lymphocytes. The mean fluorescence intensity of cytokines produced by CD4^+^ T lymphocytes from each organ was evaluated at two different moments of tumor progression; Day 17: tumor colonization in the breast draining lymph nodes, spleen, and lungs; Day 23: metastasis in all organs evaluated. **(A)** Principal component analysis (PCA) during tumor progression. Left panel: naïve (tumor free) PCA; Middle panel: Day 17 PCA; Right panel: Day 23 PCA. **(B)** PCA of each organ during tumor progression. Created with BioRender.com.

To characterize a signature of CD4+ T lymphocytes in different organs, flow cytometry of cytokines produced by CD4+ T lymphocytes was performed in the inguinal lymph node, spleen, lung, bone marrow, and liver. According to the results of the Principal Component Analysis (PCA), we observed that in steady-state mice (naïve group) the profile of cytokines produced by CD4+ T lymphocytes is unique, considering that there is no overlapping of polygons among the organs evaluated (**Figure 6A**, PCA on the left). To identify whether this signature was maintained during tumor growth, we used the 4T1 murine breast tumor model and, again, evaluated the cytokines produced by CD4+ T lymphocytes, in the same organs, which are target organs of metastasis in the model. We demonstrated that, as in steady-state, there is no overlap of the polygons in the groups at D17 and D23 (**Figure 6A**, middle and right PCA respectively). Stratifying each organ throughout tumor progression, it is clear that the naïve and D23 groups are closer to each other, in relation to D17 in all organs analyzed. Based on the concept of tumor immunoediting, we hypothesized that at D23 it is in the equilibrium/evasion phases, justifying the proximity between the phenotypes of CD4+ T lymphocytes and the naive moment. However, at D17, the elimination phase is likely to be predominant, with effective tumor recognition and greater production of cytokines by CD4+ T lymphocytes (**Figure 6B**).

### Final considerations

In the present review, we have compiled that even if some subtypes of T cells overlap in some organs, the role of these cells is unique and interacts with the cells’ residence in each tissue microenvironment. In a steady state, lymphocytes residing in tissues must not disrupt the tissue’s equilibrium, allowing local alterations to return to the original state and maintain tissue health. As such, the initial response is already influenced by the baseline cytokine environment specific to that organ. In infectious diseases, pathogens can invade the body through diverse routes and face unique microenvironments where different cells reside and interact. In this sense, studies have shown that the immune response of CD4^+^ T lymphocytes is impacted by the route of infection used, denoting that local infection generates different T cell phenotypes than systemic infection (291, 292), making it clear that the tissue microenvironment is determinant for the type of T CD4 response generated. When infection is controlled, the baseline response in that organ will need to be reestablished to the healthy levels, keeping with tissue homeostasis.

Unlike the response to pathogens, the immune response to cancer is very particular. The tumor cell belongs to the organism itself and is initially generated in a tissue-specific way, so some impact on the local immune response will occur. Investigating tumor-infiltrating lymphocytes is essential to developing immunotherapies and the prediction of their clinical responses with regard to the link between CD4^+^ T lymphocytes phenotype and malignancy.

In this sense immunotherapies represent a diverse set of promising therapeutic strategies that encompass different targets within the tumor immune response. For example, immune checkpoint blockade (ICB) therapy is an intervention that aims to rescue T-cell dysfunction in cancer to increase the effectiveness of antitumor immune responses (293). In this context, CD8^+^ T lymphocytes are the main focus of investigations into the mechanisms and impacts of this therapy, as they are the cells most studied regarding the phenomenon and (dys)function in the exhausted phenotype and, in addition, these cells are capable of acting directly in the elimination of tumor cells through cytotoxic mechanisms (294). However, like other types of immunotherapy, ICB is not targeted to specific cells and therefore several components of the TME are affected directly and indirectly by these therapies. In relation to CD4^+^ T lymphocytes, these cells also present an exhausted phenotype in different tumor types and, most likely, undergo modifications evoked by ICB (295), that in turn affect the antitumor immune response. As already discussed, CD4^+^ T lymphocytes and the TME influence each other, directly and indirectly, providing a complex and interdependent interaction in each type of tumor. However, attention to the impacts of these immunotherapies on the phenotypic and functional modulation of CD4^+^ T lymphocytes have only recently begun to be adequately appreciated. Even though it is an incipient investigation, of great complexity and interest and with the prospect of generating high-impact data on immunotherapies, these field of investigation was recently revisited by Saillard M (296) and Tay RE (297).

Although it represents a significant advance in oncological treatment, immunotherapy continues to demonstrate efficacy in a minority of patients, and even those who initially respond favorably may eventually develop resistance (298). Resistance to immunotherapy emerges through various mechanisms, from the expression and presentation of antigens to the metabolism and nutrient availability within the TME. Tumor cells have an altered metabolism characterized by a preference for anaerobic glycolysis to produce energy, even in the presence of oxygen, known as the Warburg effect. Consequently, the TME is hypoxic, acidified and nutritionally depleted, conditions common to solid tumors of different origins (299). However, such characteristics impact the local immune response, for example, hypoxia can indirectly induce the expression of PD-L1in tumor cells, a ligand for the PD-1 immune checkpoint, contributing to T cell inhibition (300). Furthermore, tumor cells can compete with immune cells for essential nutrients in the TME, such as glucose, limiting availability to T cells and compromising their effector phenotype/function (301). Therefore, combining immunotherapy with targeted metabolic interventions could serve as a potential therapeutic strategy.

With this review, we highlight the role of tissue specific CD4^+^ T lymphocytes in homeostasis and also in tumor establishment/development across different organs. Since CD4^+^ T lymphocytes play a central role in the regulation of the antitumor immune response, the presence and function of these cells are essential for the coordination and amplification of the adaptive immune response, particularly in specific tumor tissues, indicating the potential role of indicators of prognosis and response treatment of these cells.

## Methods

### Animal model

The 8- to 12-week-old female BALB/c J mice used in this study. All animal procedures were approved by the Ethical Committee for Animal Experimentation (L0024/21). The syngeneic murine breast tumor model with 4T1 was performed as described previously (302). Distant metastases were detected in inguinal lymph nodes, spleen, lung and liver at day 17 and bone marrow at day 23. We inoculated anti-CD90.2 (1ug/100uL per mouse) to exclude circulating lymphocytes (303). The organs were then processed and stained for CD4, TNF-α, IFN-y, IL-13, IL-17A, IL17-F and GM-CSF. All samples were acquired using a FACSCanto (BD Biosciences, CA). Acquisitions were performed using BD FACSDiva. Analyses were performed using FlowJo software (BD Biosciences, CA).

### Principal component analysis

Principal component analysis was calculated using as input data the mean fluorescence intensity (MFI) of fluorophores conjugated to antibodies used in the detection of intracellular cytokines. The analysis was carried out in R (4.3.2) using the prcomp function from the stats package (4.3.2) with the center and scale arguments as TRUE. The values obtained were used to plot the graphs using the ggplot2 (3.4.4) and ggfortify (0.4.16) packages. The layout of the graphics was done in BioRender.

## Data availability statement

The original contributions presented in the study are included in the article/supplementary materials. Further inquiries can be directed to the corresponding author.

## Ethics statement

The animal study was approved by Comissão de Ética no Uso de Animais do Instituto Oswaldo Cruz (CEUA-IOC) - L-024/2021. The study was conducted in accordance with the local legislation and institutional requirements.

## Author contributions

MP: Writing – review & editing, Writing – original draft, Visualization, Investigation, Conceptualization. RG: Writing – review & editing, Writing – original draft, Visualization, Formal analysis. TG-S: Writing – review & editing, Writing – original draft. ZV: Writing – review & editing. AB: Writing – review & editing, Supervision, Funding acquisition, Conceptualization.
